# Real-time dosimetry in interventional radiology – comparing the occupational radiation exposure in fluoroscopy-guided lower extremity and abdominal procedures

**DOI:** 10.1007/s00330-025-11566-5

**Published:** 2025-04-13

**Authors:** Kristina Krompaß, Mareike Mutschler, Jan-Peter Grunz, Annette Thurner, Thorsten Alexander Bley, Wolfram Voelker, Ralph Kickuth

**Affiliations:** 1 Department of Diagnostic and Interventional Radiology, University Hospital Würzburg, Würzburg, Germany; 2 Department of Radiology, University of Wisconsin–Madison, Madison, Wisconsin USA; 3 Department of Medicine I/Cardiology, University Clinic of Würzburg, Würzburg, Germany

**Keywords:** Real-time dosimetry, Radiation dose, Digital subtraction angiography, Scatter radiation, Radiation protection

## Abstract

**Objective:**

Radiation safety concerns have spurred the development of real-time dosimetry systems. This study investigated the occupational dose exposure of interventional radiologists during lower extremity and abdominal procedures.

**Materials and methods:**

Real-time dosimetry was performed during 102 consecutive interventions (51 lower extremity, 51 abdominal). Radiation protection measures included protective glasses (lead equivalent 0.5 mm), thyroid shielding (0.5 mm), vests (0.35 mm), aprons (0.25 mm), as well as movable acrylic and table shields (both 0.5 mm) during all procedures. Dosimeters were attached to the interventionalist’s glasses on the side of the x-ray tube, to the back of the supporting hand, and under the vest. Using standardized values over time to account for exposure time differences between interventions, dose-area products and the dose equivalent H_P_(10) were recorded in all three positions.

**Results:**

Lower extremity angiographies were associated with a substantially lower median dose-area product (5.3 vs. 51.4 Gy × cm^2^) and exposure time (462 vs. 762 s) than abdominal interventions (both *p* < 0.001). For lower extremity procedures, H_P_(10) per minute recorded by the hand, cranium/eye lens, and body trunk dosimeters was 2.45, 0.01, and < 0.01 µSv/min, respectively. Markedly higher dose equivalents were documented for the hand (7.54 µSv/min), cranium/eye lens (0.26 µSv/min), and body trunk (0.04 µSv/min) during abdominal interventions (all *p* < 0.001).

**Conclusion:**

Real-time dosimetry confirmed sufficient radiation protection with the application of dedicated safety measures, even in dose-intensive abdominal procedures. Interventionalists’ supporting hands are subjected to the highest radiation exposure, followed by the cranium/eye lens and the body trunk.

**Key Points:**

***Question*** *Active dosimetry facilitates real-time assessment of radiation exposure in different measurement sites, but a multi-dosimeter setup has not been explored for interventional radiology so far.*

***Findings*** *Occupational radiation exposure is considerably higher in abdominal than in lower extremity procedures. Interventionalists’ supporting hands receive the highest dose equivalents regardless of procedure type.*

***Clinical relevance*** *Dose monitoring in real time is key to understanding the radiation burden of different anatomical features during image-guided interventions. Especially in dose-intensive abdominal procedures, protective measures are essential to minimize the occupational radiation exposure of the interventionalist.*

**Graphical Abstract:**

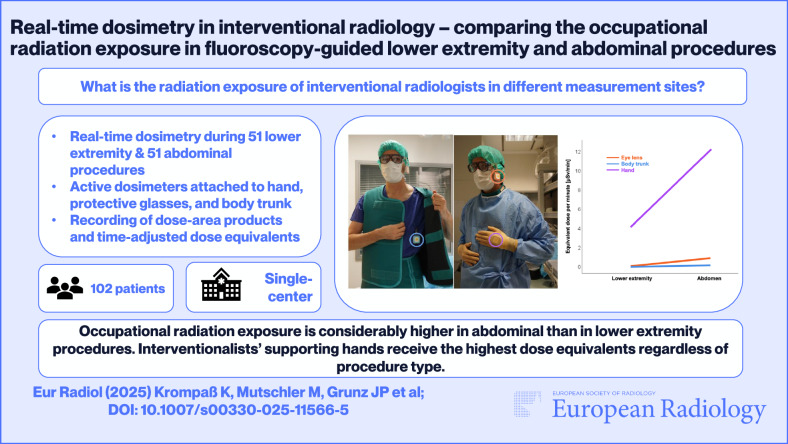

## Introduction

Interventional radiology nowadays offers a safe and effective alternative for many surgical procedures [1–3]. Minimally invasive techniques, such as percutaneous transluminal angioplasty (PTA), have been shown to reduce the mortality of patients, hospital time, and costs compared to conventional surgery [4, 5]. This is particularly important for older and multimorbid patients, whose numbers are steadily growing due to demographic change [6]. Unsurprisingly, the number of interventional radiology procedures has increased considerably in the last decade, resulting in the continuous evolution of both radiation protection measures and dosimetry systems [7].

Since the increase in radiation exposure in the field of interventional radiology applies not only to patients but also to the radiologists performing interventional procedures, a growing need for personnel dosimetry of medical staff has been voiced in recent years. The risks for interventionalists, who stand in close proximity to the primary source of radiation (i.e., the x-ray tube), originate primarily from scattered radiation deflected by the patient [8]. Due to its unpredictable nature, scattered radiation represents the main risk factor for carcinogenesis in interventional radiologists [9]. While the ALARA principle is firmly anchored in radiation protection practice, keeping the dose “as low as reasonably achievable” by scan parameter optimization is not sufficient in interventional radiology, where examiners are repeatedly exposed to radiation over longer periods of time [10].

Various investigations corroborate that adequate protective measures against occupational exposure are absolutely essential for medical staff since the consequences of numerous contacts with x-ray radiation can be observed even years after exposure [11]. Among the different parts of anatomy at risk during an interventional procedure, especially the eye lens has been in the center of radiation protection efforts as of late. According to studies, the development of radiation-induced cataracts can no longer be classified as a deterministic consequence but rather as a stochastic danger [12]. Consequently, the dose limit for the eye lens in Europe has been reduced from 150 to 20 mSv in a single year or 100 mSv in any five consecutive years subject to a maximum dose of 50 mSv in a single year, following the most recent directive of the European Atomic Energy Community (i.e., 2013/59/Euratom) [13].

Although a personal dosimeter is mandatory to record occupational radiation exposure, such passive dosimetry (e.g., via film blackening or optically stimulated luminescence) has limited capabilities in the relevant low-dose range. Furthermore, over-exposure to radiation can only be detected retrospectively. Active dosimetry, on the other hand, relies on electronically based data acquisition, enabling real-time measurements of even minimal radiation doses, which provide information about the current exposure situation [14]. While the available literature is frequently based on data obtained during cardiac interventions [7], hand surgery [15], and neurosurgical procedures [16], investigations on real-time dosimetry in interventional radiology are scarce [17]. Particularly, dose assessment for radiation-sensitive anatomy, such as the eye lens, is lacking thus far.

The present study aims to close this research gap by comparing the occupational dose exposure of interventional radiologists’ cranium/eye lens, hand, and body trunk during lower extremity and abdominal procedures with multiple active dosimeters. By determining the dose equivalent in each position via real-time dosimetry, we assessed whether established measures suffice the high demands of protection against scattered radiation.

## Materials and methods

### Study concept

In this single-center investigation, we retrospectively analyzed consecutive data from lower extremity and abdominal procedures monitored by real-time dosimetry at a tertiary-care university hospital between November 2017 and December 2018. Approval of the study design was obtained from the local ethics committee (20240513 01). Patients gave their written informed consent at least 24 h before the respective intervention. Study inclusion required the use of three active dosimeters in predefined positions, while exclusion criteria were defined as follows: Change of staff during the interventional procedure, deviation from the standard radiation safety measures, and technical failure of the dosimetry system. All procedures were performed by a board-certified interventional radiologist with 27 years of experience (R.K.) and took place in dedicated angiography suites equipped with state-of-the-art angiography systems using under-table x-ray tubes (Axiom Artis Zee, Siemens Healthineers).

**Fig. 1:**
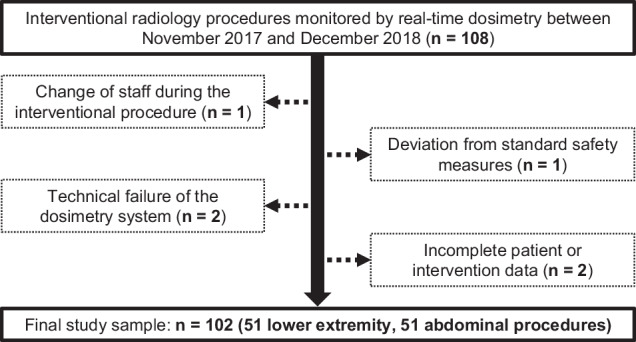
Flow chart illustrating the study inclusion and exclusion criteria as well as the final sample for data analysis

Adhering to the predefined exclusion criteria, six procedures were excluded from the final study sample, which encompassed a total of 102 interventions (Fig. 1). Of these, 51 (50%) were digital subtraction angiographies (DSA; with or without PTA) of the lower extremity using an antegrade femoral access, while the other group comprised 51 (50%) abdominal procedures. All abdominal interventions except for one transjugular liver biopsy were performed via a femoral access route with retrograde arterial or antegrade venous catheterization. The spectrum included DSA with or without PTA, transarterial chemoembolization, prophylactic embolization prior to transarterial radioembolization, and adrenal vein sampling, among others. For each of the 102 patients in the investigated sample, sex, age (years), height (m), and weight (kg) were recorded. Subsequently, specific body mass indices (BMI) were calculated for each patient and individuals were grouped into different BMI clusters: Underweight (< 18.5 kg/m^2^), normal weight (18.5–24.9 kg/m^2^), pre-obesity (25–29.9 kg/m^2^), obesity grade I (30–34.9 kg/m^2^), obesity grade II (35–39.9 kg/m^2^), and obesity grade III (> 40 kg/m^2^).

### Radiation dose analysis

For all interventions, an iodine-based contrast agent (Imeron 300; Bracco Imaging) was administered in combination with intermittent fluoroscopy and DSA to visualize vessel anatomy. Dose-area products (Gy × cm^2^) and exposure time data (seconds) were retrieved from the patient dose report automatically generated by the scanner. Standardized radiation protection measures during procedures included the use of protective glasses (lead equivalent of 0.5 mm), thyroid shielding (0.5 mm), vest (0.35 mm), and skirt (0.25 mm) by the interventional radiologist. A lead curtain (0.5 mm) was attached to the patient table on the side of the examiner. Furthermore, a movable lead acrylic shield (0.5 mm) was positioned between the interventional radiologists and the patient whenever possible (Supplementary Table S1).

**Fig. 2:**
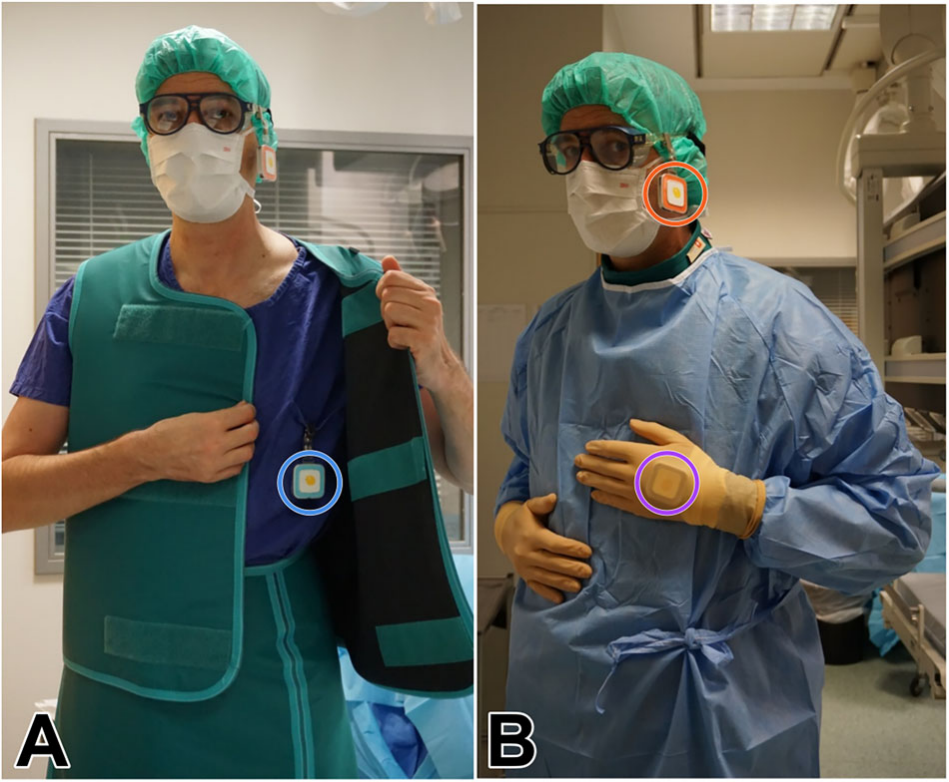
Three real-time dosimeters were attached to the interventional radiologist before each procedure: First, the blue dosimeter was placed underneath the protective vest (**A**). Second, an orange dosimeter was attached to the side of the protective glasses facing the radiation source. Finally, the purple dosimeter was positioned under the sterile glove on the back of the supporting hand, i.e., the hand that is closer to the introducer sheath during the intervention (**B**)

In order to estimate the occupational dose exposure of the cranium/eye lens, hand, and torso, real-time dosimeters (RaySafe i2, Unfors RaySafe AB) were attached to the interventional radiologist’s glasses on the side of the x-ray tube, to the back of the supporting hand (i.e., the hand which is closer to the introducer sheath during the intervention), and under the protective vest, respectively (Fig. 2). The employed dosimetry system has a dose rate range of 40 µSv/h–300 mSv/h and a dose-response time of < 1 s above 100 µSv/h and of < 5 s otherwise. It measures the dose equivalent quantity H_P_(10), which represents the dose received at a 10 mm depth from the skin [18]. To account for exposure time differences between interventions, standardized dose quantities over time were computed. Real-time dosimetry facilitates the immediate transfer of radiation data to a central hub, where the dose exposure is visualized in the form of bar charts using dedicated viewer software (Dose Manager, Unfors RaySafe AB).

### Statistical analysis

Dedicated software (SPSS Statistics v29.0; IBM) was employed for statistical analyses. Continuous variables were tested for normal distribution using the Shapiro–Wilk test. For normally distributed items, mean ± standard deviation is given. Without normal distribution, absolute frequencies and percentages are reported instead with median values and interquartile ranges. For comparisons between lower extremity and abdominal interventions, either *t*-tests (in continuous data with normal distribution) or Friedman and Mann–Whitney U tests were employed. To illustrate the correlation between dose-area products and the measured scattered radiation, Pearson coefficients (*r*) were computed. Null hypotheses were rejected if the corresponding *p*-value was ≤ 0.05.

## Results

### Intervention and patient characteristics

In total, 102 patients (58 men, 69.8 ± 12.3 years) were included in the statistical analysis. The majority of patients were between 61 and 80 years old. Among the two subgroups, patients undergoing lower extremity procedures were substantially older than individuals in the abdominal intervention sample (75.9 ± 9.4 vs. 63.7 ± 11.8 years; *p* < 0.001). In contrast, no significant difference was ascertained regarding patient height (168.5 ± 7.5 vs. 170.4 ± 9.1 cm; *p* = 0.21), weight (75.9 ± 14.5 vs. 80.3 ± 16.4 kg; *p* = 0.12), and BMI (26.3 ± 5.2 vs. 27.2 ± 5.1 kg/m^2^; *p* = 0.21). In both samples, most patients were in the pre-obesity group (41.2% of lower extremity vs. 47.1% of abdominal interventions). Detailed patient characteristics are provided in Table 1.

**Table 1:** Patient samples

Parameters	Lower extremity procedures	Abdominal procedures	Overall
Number of interventions	51 (50%)	51 (50%)	102 (100%)
Sex (men/women)	27/24 (52.9%/47.1%)	31/20 (60.8%/39.2%)	58/44 (56.9%/43.1%)
Age (mean ± SD) (years)	75.9 ± 9.4	63.7 ± 11.8	69.8 ± 12.3
≤ 40 years	0 (0%)	2 (3.9%)	2 (2%)
41–60 years	3 (5.9%)	16 (3.1%)	19 (18.6%)
61–80 years	31 (60.8%)	28 (54.9%)	59 (57.8%)
> 80 years	17 (33.3%)	5 (9.8%)	22 (21.6%)
Body mass index (mean ± SD) (kg/m²)	26.3 ± 5.2	27.2 ± 5.1	26.7 ± 5.1
Underweight (< 18.5 kg/m²)	3 (5.9%)	2 (3.9%)	5 (4.9%)
Normal weight (18.5–24.9 kg/m²)	15 (29.4%)	12 (23.5%)	27 (26.5%)
Pre-obesity (25–29.9 kg/m²)	21 (41.2%)	24 (47.1%)	45 (44.1%)
Obesity grade I (30–34.9 kg/m²)	7 (13.7%)	8 (15.7%)	15 (14.7%)
Obesity grade II (35–39.9 kg/m²)	4 (7.8%)	5 (9.8%)	9 (8.8%)
Obesity grade III (> 40 kg/m²)	1 (2%)	0 (0%)	1 (1%)

If not otherwise specified, data is reported as absolute numbers with relative frequencies in parentheses

*SD* standard deviation

In the lower extremity procedure group, DSA with PTA was the predominant procedure at 92.2%. Meanwhile, a large variety of intervention types were included in the abdominal sample with transarterial chemoembolization (39.2%) and adrenal venous sampling (29.4%) being most common. The detailed frequency of each procedure is displayed in Table 2.

**Table 2:** Distribution of intervention types

Type of intervention	Frequency
Lower extremity procedures	51
Diagnostic digital subtraction angiography	4 (7.8%)
Percutaneous transluminal angioplasty	47 (92.2%)
Abdominal procedures	51
Transarterial chemoembolization	20 (39.2%)
Adrenal venous sampling	15 (29.4%)
Percutaneous transluminal angioplasty	6 (11.8%)
Selective internal radiation therapy	5 (9.8%)
Embolization	3 (5.9%)
Transjugular liver biopsy	1 (2%)
Diagnostic digital subtraction angiography	1 (2%)

Data is reported as absolute numbers with relative frequencies in parentheses

### Radiation dose

The median exposure time over all interventions was 525 s (interquartile range 378–941 s), while the recorded dose-area product amounted to 18.3 Gy × cm^2^ (5.3–51.2 Gy × cm^2^). In direct comparison of lower extremity and abdominal procedures, the latter were associated with a considerably longer exposure time (462 [258–618] vs. 762 [462–1200] seconds; *p* < 0.001) and an almost tenfold higher dose-area product (5.3 [3.6–7.6] vs. 51.4 [39.4–111.4] Gy × cm^2^; *p* < 0.001).

**Fig. 3:**
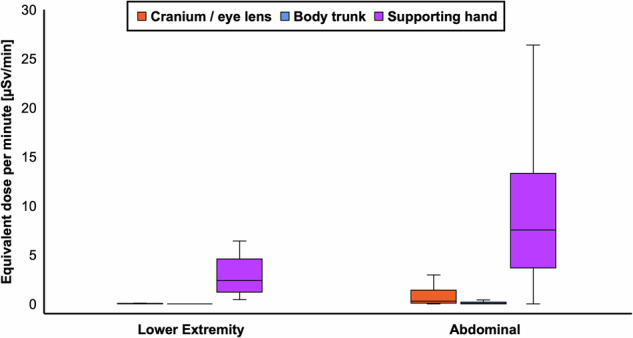
While the dose equivalents recorded by the cranium/eye lens (orange) and body trunk dosimeters (blue) were rather low in all examinations, the interventionalist’s supporting hand (purple) showed a substantially higher dose exposure, which increased considerably in dose-intensive abdominal procedures with longer examination times

Based on standardized assessment via real-time dosimetry, the radiation exposure recorded for the supporting hand, the cranium/eye lens, and the body trunk of the interventional radiologist during lower extremity angiographies was 16.28, 0.08, and < 0.01 µSv, respectively, whereas the time-corrected dose equivalents for the three measurement sites were 2.45, 0.01, and < 0.01 µSv/min. Regardless of the dosimeter position, higher absolute and time-corrected doses were documented during abdominal procedures (all *p* < 0.001). The differences between lower extremity and abdominal interventions are visualized in Fig. 3. Image-guided procedures in the abdomen were associated with doses of 109.11, 4.81, and 0.46 µSv for the dosimeters recording the radiation exposure of the supporting hand, the cranium/eye lens, and the body trunk. Performing time correction, the respective measurement sites were exposed to a dose equivalent per minute of 7.54, 0.26, and 0.04 µSv/min. Measures of radiation dose are summarized in Table 3.

**Table 3:** Exposure time and radiation dose

	Exposure time [s]	Dose-area product [Gy × cm^2^]	Dose equivalent H_P_(10) [µSv]	Dose equivalent over time [µSv/min]
Lower extremity procedures				
Cranium/eye lens	462 (258–618)	5.3 (3.6–7.6)	0.08 (0.02–0.36)	0.01 (< 0.01–0.04)
Body trunk			< 0.01 (< 0.01 to < 0.01)	< 0.01 (< 0.01 to < 0.01)
Hand			16.28 (10.17–33.45)	2.45 (1.21–4.55)
Abdominal procedures				
Cranium/eye lens	762 (462–1200)	51.4 (39.4–111.4)	4.81 (0.78–12.85)	0.26 (0.08–1.25)
Body trunk			0.46 (0.04–2.51)	0.04 (< 0.01–0.17)
Hand			109.11 (49.82–143.49)	7.54 (3.86–13.13)
Overall				
Cranium/eye lens	525 (378–941)	18.3 (5.3–51.2)	0.44 (0.06–5.92)	0.06 (0.01–0.41)
Body trunk			< 0.01 (< 0.01–0.46)	< 0.01 (< 0.01–0.04)
Hand			35.83 (14.45–120.56)	4.42 (1.92–10.35)

Data is reported as median values with interquartile ranges in parentheses. H_P_(10) = dose received at 10 mm depth from the body surface

## Discussion

In this study, we retrospectively analyzed real-time dosimetry data from 102 interventions to compare the occupational radiation exposure of interventional radiologists during lower extremity and abdominal procedures. While abdominal interventions were generally associated with longer exposure times and higher overall radiation dose, our results indicate that the supporting hand of the examiner is exposed to the highest radiation dose by far, regardless of a procedure’s target region. By also determining the dose equivalents for the cranium/eye lens and body trunk in a three-dosimeter setup, we provide evidence that the high demands of protection against scattered radiation in interventional radiology can be met when applying dedicated safety measures.

As a major finding, we were able to show that the doses from scattered radiation are in the nano- to microsievert range in each of the three investigated anatomical positions. Although radiation exposure was higher in abdominal procedures compared to lower extremity interventions, the annual dose limits of 20 mSv/year for the eye lens and 500 mSv/year for the hand [13, 19], do not constitute a problem if radiation protection is used consciously. The average dose recorded by the cranium/eye lens dosimeter, for example, was merely 0.08 µSv for lower extremity and 4.81 µSv for abdominal interventions in the present study, hence a total of 250,000 leg or 4158 abdominal procedures could be performed per year before surpassing the permitted exposure limit. The observed dose equivalents were in line with previous phantom-based investigations [20].

The radiation exposure of hospital staff in the angiography suite is directly proportional to the dose a patient receives, hence the examiner’s exposure is generally higher during longer procedures or in adipose patients. In the investigated sample, the radiation exposure time associated with abdominal procedures was approximately 65% longer compared to lower extremity procedures, whereas no significant BMI difference was ascertained between the two patient groups. Since this time difference distorts the comparability of interventions, the item “dose equivalent per minute” was introduced for the present study. Notably, the time-corrected radiation exposure recorded by the cranium/eye lens and hand dosimeters was approximately 26 times and three times higher in abdominal procedures. Due to the very low H_P_(10) measured by the body trunk dosimeter underneath the protective vest, comparability of absolute numbers is limited.

Being characterized by relatively high dose rates and short pulse duration, pulsed radiation fields are the standard in interventional radiology. Ankerhold et al stated in 2009 that the characteristics of active dosemeters determined in a continuous radiation field cannot be transferred to pulsed fields [21]. While this statement remains correct, the active dosimetry system employed in this investigation has been designed for dedicated use in interventional radiology and cardiology departments. Multiple studies have substantiated the applicability of active dosimetry systems, such as RaySafe i2, for real-time dose monitoring of pulsed radiation fields. Baptista et al investigated a sample of 41 cardiologic interventions, where a cumulative effective dose of 458 µSv was ascertained for the examiner. However, in contrast to the three active dosimeters used in the present study, the authors relied on a single dosimeter attached to the outside of the protective vest [22]. A similar dosimeter position was chosen by Kim et al for the neurosurgical treatment of cerebral aneurysms in a small patient sample of 19 individuals, establishing a dose equivalent of 10 µSv per procedure with standard protective measures [16]. Koch et al employed the single-dosimeter setup in 214 interventional radiology procedures, observing a considerable reduction in operator radiation dose and exposure time [17]. Another investigation of real-time dosimetry with one over-the-vest dosimeter was performed during 94 hand surgery procedures by van Rappard et al [15]. While the study employed a mini C-arm scanner with ten times lower tube current, decreasing comparability to our investigation due to different scattered radiation behavior, it must also be acknowledged that the use of x-rays in hand surgery is much more limited, indicated by the median fluoroscopy time of only 11 s [23]. Although the vendor recommends the use of RaySafe i2 dosimeters outside the protective gear, it must be acknowledged that measurements above the vest do not reflect the actual radiation exposure of the interventional radiologist. To investigate the occupational dose in a realistic clinical scenario, the effect of standardized protective measures (such as the vest) was taken into account in the present study. However, due to the very low H_P_(10) measured by the body trunk dosimeter, comparability of absolute numbers is limited, and results should be interpreted with caution.

Measurement uncertainty is a highly relevant topic in real-time dosimetry. The vendor of the RaySafe i2 dosimetry system describes an x-ray dose uncertainty of 5% or 1µSv [24]. Considering its dose rate range of 40 µSv/h–300 mSv/h, the dosimeter may miss some lower doses in the presence of optimized protective equipment. Of note, Inaba et al were able to demonstrate excellent dose linearity and a dose-rate dependency below 5% for reasonably applicable dose rates in an earlier study [25]. The energy dependency of the employed system is stated to be ± 20% within the N40–N100 range (effective 33–83 keV) and ± 30% within the N100–N120 range (effective 83–101 keV) [18]. Angular dependency should also not be underestimated in interventional radiology, since both the beam angulation and the examiner’s position within the angiography suite can differ considerably over the course of an intervention. Regarding the influence of the x-ray beam angle, the manual states a dose-response variability of ± 5% for 5°, ± 30% for 50%, and +200%/−100% for 90° [18], which is considerable given the unplannable nature of image-guided procedures, especially in emergency situations.

Negative effects of radiation at the cellular level, such as carcinogenesis, have been demonstrated in numerous investigations [26, 27]. Since interventional radiologists are repeatedly exposed to low-dose scattered radiation on a daily basis, particular protective measures are of utmost importance for these individuals. Future radiation protection efforts should concentrate on developing smaller active dosimeters that can be integrated seamlessly into the personal protective gear of surgeons and interventionalists. Thereby, the willingness to wear multiple real-time dosimeters simultaneously could be increased. In light of the negative effects of repeated radiation exposure to various tissues, wearing one passive dosimeter with monthly readout should be questioned by any medical professional performing x-ray-guided procedures at this point in time. Ease of use and better availability of active dosimetry systems would render the extensive data collection less costly and time-consuming. Prospective studies could further evaluate the effect of radiation hygiene training with various real-time dosimeters during routine interventions.

On a general note, interventional radiologists should adhere to the ALARA principle, meaning that they should try to keep the dose for each procedure as low as reasonably achievable. Applying less radiation during an intervention is by far the most effective way to lower one’s own exposure. This can be achieved, for example, by shortening the examination time, performing fluoroscopy at a low pulse rate, and applying appropriate beam collimation. In addition to the use of dedicated safety measures, such as protective clothing and shields, the examiner should be aware of the fact that radiation exposure is inversely proportional to the square of the distance. While interventional radiologists stayed with the patient at all times during each of the procedures included in this study, stepping aside or leaving the room in appropriate situations could also reduce radiation exposure.

Several methodological limitations have to be acknowledged with regard to the study design. First, real-time dosimetry was exclusively performed for the interventional radiologist leading the procedure. Dose monitoring of other medical staff in the angiography suite should be a focus of future research. Since only data from one highly specialized interventional radiologist was analyzed, results may have been different for other medical staff with less experience. Despite the interventionalist’s experience, we must assume that his hands were occasionally directly within the x-ray beam. Especially antegrade femoral access routes in obese patients can be difficult to establish, potentially leading to direct radiation exposure of the hands. Second, only limited conclusions can be drawn about the dose reduction effect of each protective measure, since all were applied simultaneously, and no procedures were performed without radiation protection equipment. Third, as the real-time dosimeter for the cranium was attached to the side of the protective goggles and therefore not positioned behind the lead glass, the actual eye lens dose cannot be quantified. Fourth, the employed real-time dosimetry system does not discern the radiation exposure from fluoroscopy and DSA. Fifth, the system can only measure the deep dose equivalent H_P_(10), which is not the primary option for eye lens and hand dosimetry. For these measurement sites, obtaining H_P_(3) and H_P_(0.07) at 3 and 0.07 mm from the body surface would have been desirable. Finally, only one dosimetry system and one C-arm system, each from a single vendor, were used in this investigation, limiting the inclusion time period and potentially the transferability of results.

In conclusion, real-time dosimetry allows for ubiquitous monitoring of the dose exposure in interventional radiology, confirming sufficient protection against scatter radiation with the application of dedicated safety measures even in dose-intensive abdominal procedures. Regardless of target region, the supporting hand of interventional radiologists is by far subjected to the highest radiation exposure, followed by the cranium/eye lens and the body trunk.

## Supplementary information

ELECTRONIC SUPPLEMENTARY MATERIAL

## Abbreviations

DSA: Digital subtraction angiography
PTA: Percutaneous transluminal angioplasty
