# Supplementary material for: Real-time dosimetry in interventional radiology – comparing the occupational radiation exposure in fluoroscopy-guided lower extremity and abdominal procedures
Source: Eur Radiol. 2025 Apr 13;35(10):6575–82. doi: 10.1007/s00330-025-11566-5 (PMC12417226; doi:10.1007/s00330-025-11566-5)
Supplement: Supplementary file 1 — ELECTRONIC SUPPLEMENTARY MATERIAL [file 330_2025_11566_MOESM1_ESM.pdf]

Real-time dosimetry in interventional radiology – Comparing the occupational radiation exposure in fluoroscopy-guided lower extremity and abdominal procedures

ELECTRONIC SUPPLEMENTARY MATERIAL

Supplemental Table S1 – Radiation protection measures

Type of protection	Manufacturer	Model	Lead equivalent [mm Pb]
Protective glasses	Mavig GmbH, Munich, Germany	Eurolite, Z87	0.5 – 0.75
Thyroid shield	Scanflex Medical AB, Täby, Sweden	7351LL	0.5
Vest	Scanflex Medical AB, Täby, Sweden	61323LL063	0.5
Apron	Scanflex Medical AB, Täby, Sweden	62223LL068	0.35
Movable acrylic shield	Mavig GmbH, Munich, Germany	OT25B050	0.5
Lower body radiation and top shield	Kenex, Harlow, England	312/DS-039/1	0.5
